# Afferent loop syndrome 7‐years post Roux‐en‐Y gastrojejunostomy: An often‐forgotten pancreatitis cause. A case report

**DOI:** 10.1002/ccr3.8627

**Published:** 2024-05-14

**Authors:** Vivien Nguyen, Goutham Sivasuthan

**Affiliations:** ^1^ Department of General Surgery Logan Hospital Meadowbrook Queensland Australia

**Keywords:** Afferent loop syndrome, bariatric surgery, Billroth II gastrojejunostomy, pancreatitis, post‐operative complication, Roux‐en‐Y gastrojejunostomy

## Abstract

Afferent loop syndrome is a rare post‐operative complication following upper gastrointestinal bypass surgeries, usually occurring within the first two weeks post‐operation. This case report, however, outlines afferent loop syndrome almost a decade post‐surgery, which was managed conservatively. A 54‐year‐old woman presented with a few days' history of epigastric pain, vomiting, and constipation. She had undergone a sleeve gastrectomy and was converted to a Roux‐en‐Y gastrojejunostomy for weight loss 9 and 7 years ago, respectively. Serum lipase was elevated at 1410 IU/L. Computed tomography showed high‐grade proximal small bowel obstruction, involving the efferent and afferent loops of the Roux‐en‐Y gastric bypass. The patient was given intravenous rehydration, electrolyte replacement and had a nasogastric tube inserted. She was discharged on day 5 of admission without significant sequelae. Afferent limb syndrome should be considered in patients with altered upper gastrointestinal anatomy who present with pancreatitis, regardless of the time period post‐operatively. Future guidelines should further more outline the factors indicated for surgical versus conservative management.

## 1 INTRODUCTION

Afferent loop syndrome is a rare but serious postoperative complication seen in upper gastrointestinal bypass surgeries such as Billroth II gastrojejunostomy, Roux‐en‐Y gastrojejunostomy and pancreaticoduodenectomy.^1^, ^2^, ^3^ Afferent loop syndrome most commonly presents within the first 2 weeks postoperative period and the majority require urgent decompression via endoscopic placement of a nasogastric tube or surgical intervention. This case report however outlines afferent loop syndrome almost a decade post initial bariatric surgery and was managed successfully through conversative measures.

## 2 CASE HISTORY

A 54‐year‐old woman presented to an Australian public hospital emergency department with a few days' history of epigastric pain radiating to the back, associated with nausea, vomiting and no bowel movements. She had previously undergone a sleeve gastrectomy, converted to a Roux‐en‐Y gastrojejunostomy for weight loss 9 and 7 years ago, respectively. She had no previous history of gallstone disease or autoimmune disease, and had been sober for almost a decade.

**FIGURE 1:**
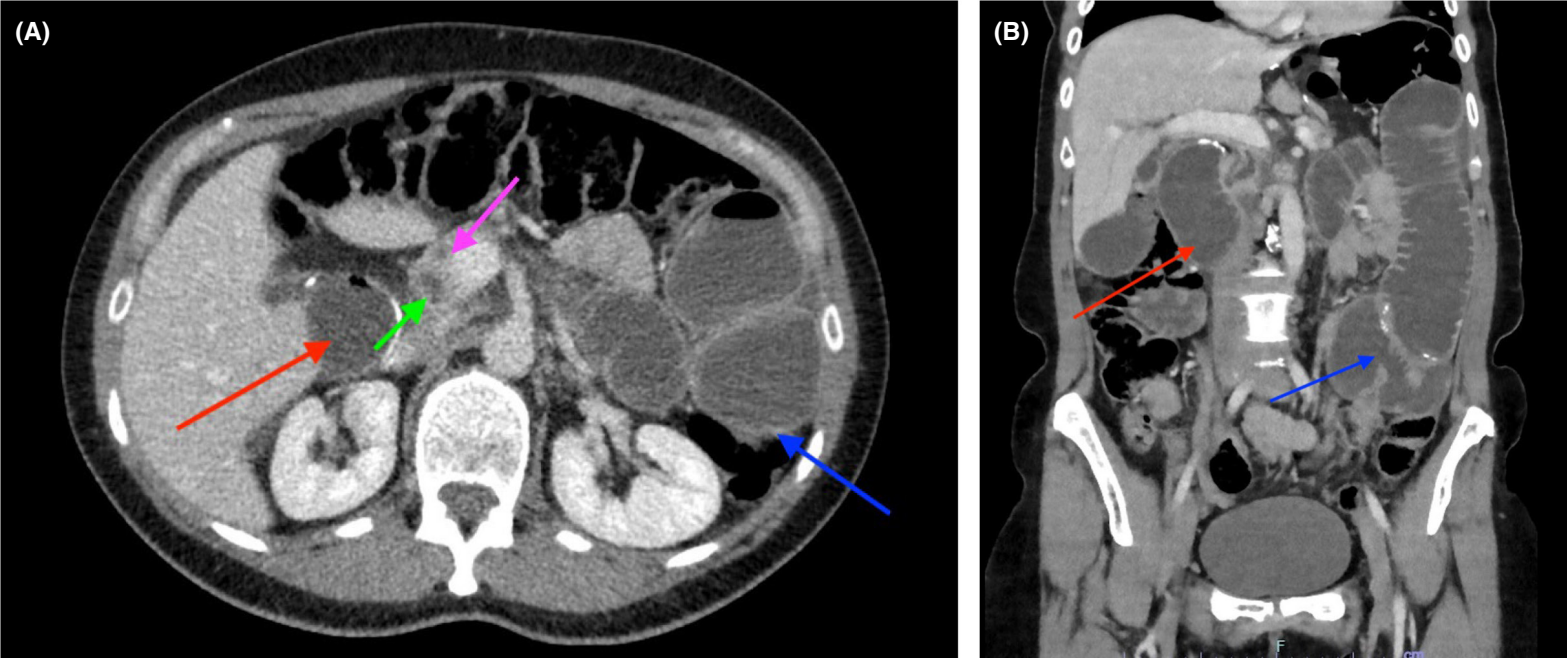
(A) Axial computed tomography of abdomen and pelvis showing a proximal small bowel obstruction, involving the efferent and afferent loops of the Roux‐en‐Y gastric bypass. Red: afferent limb; blue: efferent limb; green: common bile duct; pink: pancreatic duct. (B) Coronal computed tomography of abdomen and pelvis showing a focal transition point within the mid‐abdomen involving the small bowel, distal to the entero‐enterostomy (blue arrow). Dilated afferent limb (red arrow).

On examination, she had a grossly distended abdomen and tender epigastrium. Serum lipase was elevated at 1410 IU/L, meeting the clinical criteria for acute pancreatitis. Liver function tests and creatinine were normal on presentation. Computed tomography showed normal liver, gallbladder, kidneys and spleen, however there were features consistent with high‐grade proximal small bowel obstruction, involving both the efferent and afferent loops of the Roux‐en‐Y gastric bypass, which is diagnostic of afferent loop syndrome (Figure 1A). There was a focal transition point within the mid‐abdomen involving the small bowel, distal to the entero‐enterostomy, and also involving the distal efferent limb (Figure 1B).

## 3 METHODS

The patient was fasted, given intravenous rehydration and electrolyte replacement. A nasogastric tube was inserted and its placement confirmed on imaging. Over the course of the following day, the patient's obstructive symptoms improved with resolution of nausea, followed by later passage of bowel motions. The patient's abdominal pain also rapidly improved. She was discharged on Day 5 of admission without significant sequelae.

## 4 CONCLUSION

The patient was reviewed in the surgical outpatient clinic 3 weeks later, with complete resolution of symptoms and unremarkable ultrasound findings.

## 5 DISCUSSION

Bariatric surgery has become more prevalent with the rise in obesity.^4^ The Roux‐en‐Y gastrojejunostomy is the second most common surgery for weight loss.^4^ In patients who have undergone Roux‐en‐Y gastrojejunostomy, the biliopancreatic limb remains connected to the remnant stomach and is anastomosed distally via jejunojejunostomy.^5^ The afferent limb is the segment proximal to the anastomosis, while the efferent limb is distal to the anastomosis.^5^ Afferent loop syndrome is defined by a distal obstruction which causes distension of the afferent limb. It can arise after such upper gastrointestinal surgeries that involve anastomosis of the stomach or esophagus to the jejunum.^2^ When this occurs, there is an accumulation of bowel secretions and obstruction to the pancreaticobiliary tree, thus increasing the risk of pancreatitis, cholangitis, and significant gastric remnant distension with subsequent perforation.^1^, ^6^, ^7^

Afferent loop syndrome specifically relates to the sequalae of the afferent limb being obstructed. The significance of the afferent limb is that it is the point of biliopancreatic outflow, hence can cause biliary obstruction, cholangitis or pancreatitis as a result of stagnant flow of secretions.^2^, ^3^, ^6^ The point of obstruction can be either proximal or distal to the jejuno‐jejunostomy (the common channel anastomosis), as either can cause dilation of the afferent limb, which was the case for our patient. Intrinsic or extrinsic obstruction along the afferent limb can be caused from adhesions, internal herniation, intussusception, kinking, or malignancy.^3^ In particular, the incidence of afferent loop syndrome following Roux‐en‐Y gastrojejunostomy is ≤1.0%.^8^ This generally occurs within the first 2 weeks after surgery, however in rare instances, can present as a late complication, such as in the case for our patient, whereby adhesions may have been the precipitating factor given she had undergone two upper gastrointestinal surgeries within 2 years from one another. These aetiologies typically present with varying degrees of acuity and resulting management.^2^ Uriu et al and Gayer et al both reported cases of afferent loop syndrome 10 and 15 years postoperatively, respectively.^7^, ^9^

Acute afferent loop syndrome typically presents within the early postoperative period with sudden localized abdominal pain with nausea and vomiting, while chronic afferent loop syndrome usually occurs years after surgery with postprandial abdominal discomfort.^3^ Given that our patient's surgeries were almost a decade prior, their acute symptom presentation may be due to a sudden conversion to a complete obstruction. The nonspecific nature of these symptoms however makes diagnosis difficult, especially as concurrent pancreatitis presents in a similar manner.^3^ Nonetheless, this case report highlights that in the absence of common pancreatitis etiology (e.g., gallstones, alcohol) and a positive history of upper gastrointestinal surgery, afferent loop syndrome is a diagnosis to consider.

Alongside laboratory studies such as full blood count, liver function tests, pancreatic enzymes and serum electrolytes, abdominal imaging is necessary in the evaluation process.^3^, ^7^ Computed tomography, the gold‐standard for diagnosis, may display a fluid‐filled tubular mass with an average diameter of 5 cm, representing the obstructed afferent loop.^6^, ^7^ Distension of the intra‐ and extra‐hepatic biliary tree can arise due to increased pressure within the obstructed afferent loop.^7^ Magnetic resonance cholangiopancreatography (MRCP) is highly sensitive and specific for biliary and pancreatic duct pathology. There have been cases reported that utilized MRCP for diagnostic purposes which demonstrated the dilated afferent limb in afferent loop syndrome.^10^ Given the lack of biliary sludge or gallstones in the patient's known history, MRCP would have unlikely changed management or yielded the presence of gallstone‐related pancreatitis. MRI Pancreas can be considered as an outpatient to exclude structural abnormalities leading to pancreatitis, but would require an interval resolution of inflammatory changes to make meaningful assessment of the pancreas.

Although this case improved with conservative management, patients with afferent loop syndrome often require endoscopic nasogastric tube placement or surgical intervention.^8^ A similar case by Uriu was also successful in conservative management only, however it is unclear what indications are necessary for this to occur. Further research on the treatment approaches for acute on chronic afferent loop syndrome is required, in addition to the long‐term prognosis of cases following conservative management. Afferent limb syndrome should therefore be considered in patients with altered upper gastrointestinal anatomy who present with pancreatitis, regardless of the time period postoperatively. Future guidelines should furthermore outline the factors indicated for surgical versus conservative management.

## AUTHOR CONTRIBUTIONS

**Vivien Nguyen:** Conceptualization; writing – original draft; writing – review and editing. **Goutham Sivasuthan:** Conceptualization; supervision; writing – review and editing.

## FUNDING INFORMATION

None.

### CONSENT

Written informed consent was obtained from the patient for publication of this case report and accompanying images. A copy of the written consent is available for review by the Editor‐in‐Chief of this journal on request.

## Data Availability

Can be requested and provided with contact of corresponding author.
